# Cu^0^ Nanoparticles Deposited on Nanoporous Polymers: A Recyclable Heterogeneous Nanocatalyst for Ullmann Coupling of Aryl Halides with Amines in Water

**DOI:** 10.1038/srep08294

**Published:** 2015-02-06

**Authors:** John Mondal, Anup Biswas, Shunsuke Chiba, Yanli Zhao

**Affiliations:** 1 Division of Chemistry and Biological Chemistry, School of Physical and Mathematical Sciences, Nanyang Technological University, 21 Nanyang Link, Singapore 637371; 2 School of Materials Science and Engineering, Nanyang Technological University, Singapore 639798

## Abstract

Cu^0^ nanoparticles were deposited on a nanoporous polymer to develop a novel nanocatalyst (Cu-B) for carrying out Ullmann coupling of aryl halides with amines in water. Non-aqueous polymerization of a mixture of divinylbenzene and acrylic acid under hydrothermal conditions followed by the deposition of Cu^0^ nanoparticles were adopted to afford the Cu-B nanocatalyst. In order to compare the catalytic activity of the Cu-B nanocatalyst in the Ullmann coupling reactions, another nanocatalyst, Cu^0^ nanoparticle-loaded porous carbon (Cu-A), was also prepared. All the newly developed Cu^0^ nanoparticle-based nanocatalysts were thoroughly characterized using several characterization techniques. The Ullmann coupling reactions were carried out in water only with 1.35 mol% loading of Cu as catalytically active sites in Cu-B. The Cu-B nanocatalyst exhibited higher catalytic activity as compared with Cu-A, and also showed a good catalytic recyclability with a high consistence in the catalytic activity. No Cu leaching from the nanocatalyst surface and the smooth nanocatalyst recovery confirm the true heterogeneity in these catalytic reactions.

Constructing a new C-N bond (amination) by Cu-catalyzed Ullmann coupling of aryl halides with amines has received a considerable interest as the products (aryl amines) could be utilized potentially in pharmaceutical and material-based applications^1^,^2^,^3^,^4^,^5^,^6^,^7^,^8^,^9^. Most of the examples in the Ullmann amination, however, need the assistance of expensive N- or P-ligands to maintain the catalytic turnover (typically 5–20 mol%) in homogeneous reaction systems^10^,^11^,^12^,^13^,^14^,^15^. Thus, the development of heterogeneous catalysts has shown promising potential in sustainable applications, in which the catalysts can be recovered by simple filtration and reused for the next catalytic cycles with the minimization of undesired wastes. Homogeneous metal catalysts could be heterogenized by anchoring them onto the surface of inorganic solids including silica, alumina, zeolite, *etc*. Among various heterogeneous matrix, nanoporous polymers have received much attention in catalysis on account of their large surface area, high thermal and mechanical stability, and well-defined pore size distribution^16^,^17^,^18^. The tunable nanoporous polymers serve as the platforms to immobilize catalytically active sites (metal or metal oxide nanoparticles (NPs)) on their external surface as well as into the nanopores with the preservation of porous structures. The use of transition metal NPs as the catalysts in organic synthesis has emerged with a considerable interest due to their inherently large surface-to-volume ratio and tunable morphologies as compared with the bulk metals. Among them, Cu^0^ NPs have been widely employed in various organic reactions^19^. The stability and activity of metal NPs can be increased by avoiding the aggregation and leaching from the polymer networks due to a certain degree of chemical engagement (such as coordination and electron transfer) between the polymers and metal NPs.

In this work, we developed two new heterogeneous Cu^0^ nanocatalysts Cu-**A** and Cu-**B**, where Cu^0^ NPs were deposited on carbonaceous matrix and nanoporous polymer DVAC-1, respectively (Figure 1). Both of the catalysts were thoroughly characterized by wide-angle powder X-ray diffraction (XRD), transmittance electron microscope (TEM), field emission scanning electron microscope (FE-SEM), Raman spectroscopy, X-ray photoelectron spectroscopy (XPS), and N_2_ adsorption/desorption analysis. The Cu-**B** showed good catalytic performance for the Ullmann coupling reactions of aryl iodides or bromides with primary and secondary amines in aqueous solution, delivering a series of *N*-aryl amines in good yields with high catalyst turnover. To the best of our knowledge, this is the first example of utilizing nanoporous polymer-supported Cu^0^ heterogeneous nanocatalyst for the Ullmann amination^20^,^21^. This heterogeneous catalyst could be successfully recycled and reused for five consecutive catalytic cycles without Cu leaching from the reaction mixture.

## Results

### Synthesis and Characterization of Cu^0^ Nanocatalysts

**Figure 1:**
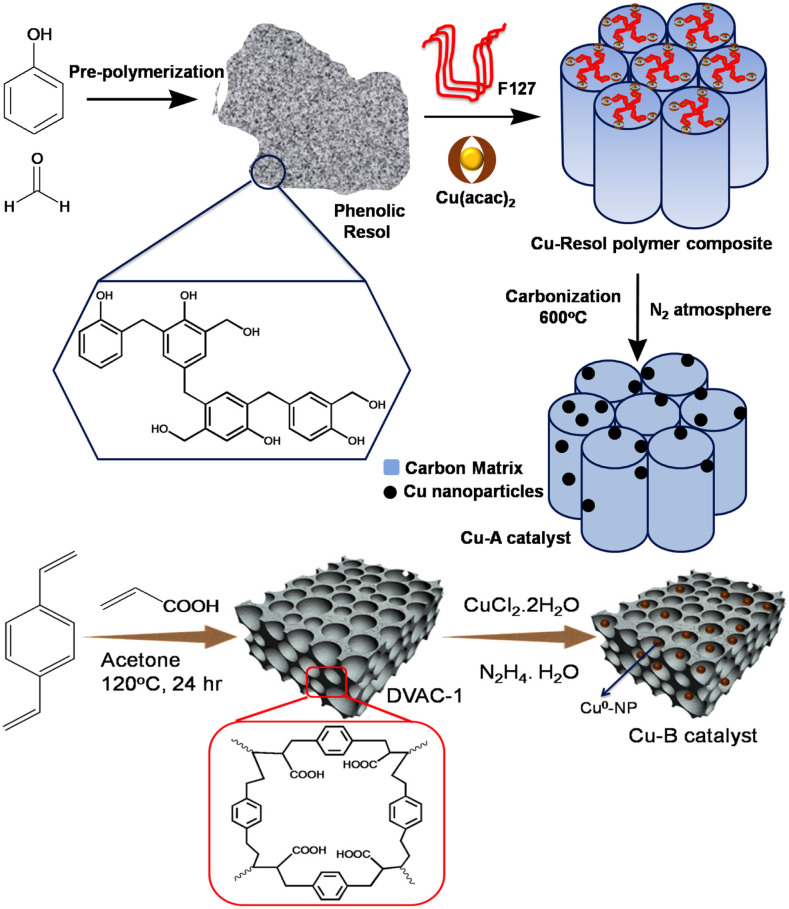
Preparation of the Cu-A and Cu-B nanocatalysts.

The catalyst Cu-**A** (Figure 1) was prepared by chelate-assisted co-assembly strategy using Cu(acac)_2_ (acac = acetylacetonate), soluble phenol formaldehyde resin and poly(ethylene oxide)-*block*-poly(propylene oxide)-*block*-poly-(ethylene oxide) triblock copolymer Pluronic F127 (M_w_ = 12600, PEO_106_PPO_70_PEO_106_, PEO = polyethylene oxide and PPO = poly propylene oxide). After thermocurring treatment, copper-complex/resol/F127 hybrid thin film was obtained. The hybrid thin film was carbonized at 600°C under an N_2_ atmosphere. During the carbonization, F127 template was decomposed, resol polymer was carbonized to generate rigid carbon matrix, and *in situ* growth of Cu^0^ NPs took place by slow decomposition of Cu(acac)_2_ complex. For the synthesis of the catalyst Cu-**B**, we used nanoporous polymer DVAC-1 developed by non-aqueous polymerization of acrylic acid. By following the synthesis route outlined in Figure 1, nanoporous organic polymer DVAC-1 was derived by non-aqueous polymerization of acrylic acid with cross linker divinyl benzene under hydrothermal condition. Azobisisobutyronitrile (AIBN) was used as the radical initiator for this reaction. The white color DVAC-1 was insoluble in water and common organic solvents. CuCl_2_•2H_2_O was utilized as the copper source. The Cu^2+^ ions were attached to the carboxylate moieties on the surface of nanoporous polymer via ligand exchange, leading to a color change from light green to dark grey. After the addition of EDA (ethylene diamine) in the reaction mixture, the color changed from dark grey to deep violet owing to the formation of Cu(EDA)_2_^2+^ complex. N_2_H_4_•H_2_O was then added to reduce Cu^2+^ ions to Cu^0^ NPs on the nanoporous polymeric network. The color of the resulted reaction mixture was deep brown, and subsequently blackish-brown Cu-**B** was obtained through precipitation.

**Figure 2:**
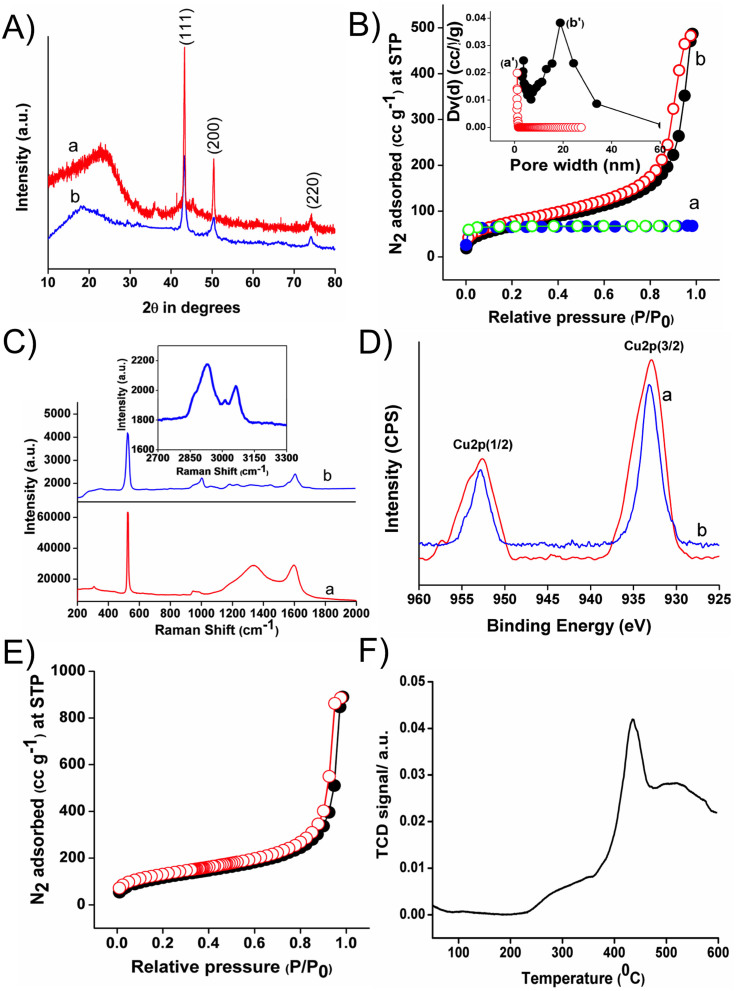
(A) Wide angle powder XRD patterns, (B) N_2_ adsorption/desorption isotherms, (C) Raman spectra, and (D) high resolution XPS spectra of (a) Cu-**A** and (b) Cu-**B** nanocatalysts. Inset of (B) shows the pore size distributions. (E) N_2_ adsorption/desorption isotherms and (F) temperature programmed desorption (TPD) of ammonia for nanoporous polymer DVAC-1.

Wide angle powder XRD patterns of Cu-**A** and Cu-**B** nanocatalysts display (Figure 2A-a and 2A-b) three well resolved peaks at 2*θ* = 43.1°, 50.3° and 74.0°, which can be readily indexed to the (111), (200) and (220) crystalline planes, respectively, corresponding to the face centered cubic (fcc) arrangement of Cu NPs (JCPDS no. 04-0836)^22^,^23^. The diffused peaks at 2*θ* = 22.7° and 18.3° for the Cu-**A** and Cu-**B**, respectively, are attributed to the amorphous frameworks of nanoporous polymer, suggesting that Cu-**A** and Cu-**B** are copper-polymer hybrids. These characteristic diffraction patterns indicate that Cu NPs are well crystallized inside the nanoporous carbon and polymer frameworks. Porous properties of these Cu nanocatalysts were established from the N_2_ sorption analysis at 77 K. N_2_ adsorption/desorption isotherms of the Cu-**A** and Cu-**B** nanocatalysts are displayed in Figure 2B. The Cu-**A** nanocatalyst exhibits a typical type I isotherm (Figure 2B-a) corresponding to the presence of micropores. The BET (Brunauer-Emmett-Teller) surface area of the Cu-**A** nanocatalyst is 90 m^2^g^−1^. Its pore size distribution (inset of Figure 2B) calculated by employing non-local density functional theory (NLDFT) reveals the presence of micropores having the diameter of 1.08 nm (Figure 2B-a′). On the other hand, the Cu-**B** catalyst shows a typical type II isotherm with a small hysteresis loop and a sharp N_2_ uptake in the high *P/P_0_* pressure region (Figure 2B-b) corresponding to the existence of interparticle void cavity^24^,^25^. The BET surface area of this catalyst is 260 m^2^g^−1^. The small hysteresis loop in the isotherm may be generated by the swelling of the polymeric network upon the gas adsorption. Its pore size distribution curve was derived by the BJH (Barrett-Joyner-Halenda) model, indicating the presence of interparticle cavity with the diameter of 18 nm (Figure 2B-b′).

The pore volumes of the Cu-**A** and Cu-**B** nanocatalysts are 0.0426 and 0.703 ccg^−1^, respectively. The pore volume of Cu-**B** is quite larger than that of Cu-**A**. The large pore volume of Cu-**B** allows for easy diffusion of reaction substrates throughout the nanoporous channels and enables them to interact with each other as well as with Cu sites for performing catalytic reaction in short reaction time^26^,^27^. Raman spectra of the two nanocatalysts are shown in Figure 2C. Raman spectrum of Cu-**A** (Figure 2C-a) displays two broad bands centered at 1337 cm^−1^ and 1597 cm^−1^ corresponding to the D band and G band, respectively, signifying the presence of amorphous carbon framework^28^. In the Raman spectrum of Cu-**B** (Figure 2C-b), the band at 1610 cm^−1^ is indicative of the existence of G band, but the peak concerning to the D band cannot be resolved properly. Two peaks appeared at 2929 cm^−1^ and 3062 cm^−1^ (inset in Figure 2C-b) for Cu-**B** could be assigned to the C-H stretching vibrations from the CH_2_ and CH_3_ groups of the polymeric framework^29^. XPS is an indispensable tool to evaluate the oxidation state of copper embedded into carbonaceous matrix and nanoporous polymer for Cu-**A** and Cu-**B**, respectively. In the XPS spectra (Figure 2D-a and 2D-b), the binding energy 2p_3/2_ centered at 932.9 eV and 933.1 eV along with other strong binding energy peaks of 2p_1/2_ at 952.7 eV and 952.8 eV for Cu-**A** and Cu-**B** were observed respectively, indicating that Cu NPs possess the oxidation state (0) inside the porous channels. The absence of satellite peaks for the 2p region also refers to the oxidation state (0) of copper^30^,^31^. No signals from Cu^2+^ were observed from the XPS spectra^32^. Nanoporous polymer DVAC-1 shows a typical type II isotherm with corresponding BET surface area of 420 m^2^g^−1^ (Figure 2E), presenting strong evidence of nanopores generated during cross-linking polymerization under hydrothermal condition. Moreover, the isotherms show that DVAC-1 adsorbs a large amount of nitrogen at high *P/P_0_* values, indicating interparticle porosity. The calculated pore volume of DVAC-1 is 1.27 ccg^−1^. Appreciable decreases in the BET surface area and pore volume of Cu-**B** as compared with nanoporous polymer DVAC-1 suggest that Cu NPs were successfully incorporated into the polymer framework. We also carried out temperature programmed desorption (TPD) of ammonia (Figure 2F) over nanoporous polymer DVAC-1 in order to calculate the loading of –COOH group in DVAC-1. DVAC-1 polymer shows a strong signal at higher desorption temperature of 434°C, corresponding to the total –COOH group loading of 1.01 mmol g^−1^.

**Figure 3:**
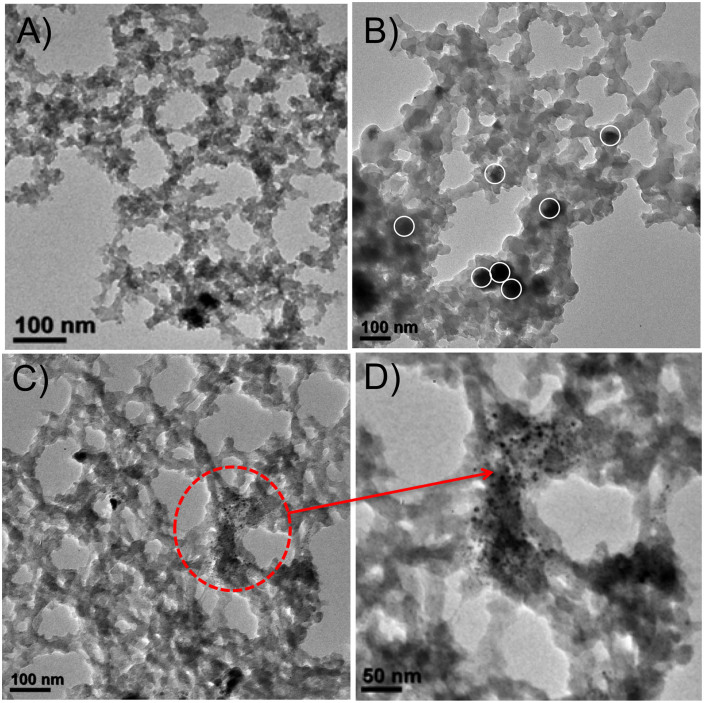
(A–D) TEM images of the Cu-B nanocatalyst. (D) is the magnified portion of (C).

**Figure 4:**
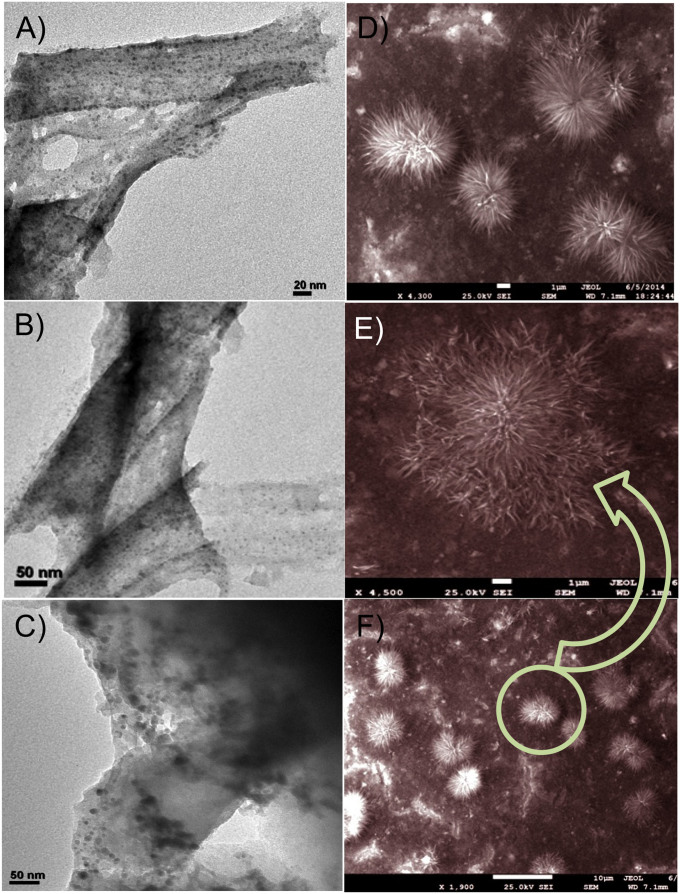
(A–C) TEM images of the Cu-A nanocatalyst and (D–F) FE-SEM images of the Cu-B nanocatalyst.

TEM analysis was performed to investigate the deposition of Cu NPs on the nanoporous polymer DVAC-1 (Figure 3). It is quite evident from Figure 3A that Cu NPs with black color were homogeneously dispersed on the surface of the conjugated polymeric network DVAC-1^33^,^34^. Individual Cu NPs marked with white circle could be clearly identified from Figure 3B, with the particle size varying from 9.8 nm to 14.3 nm. It should be mentioned that Cu NPs might undergo slight aggregation to generate copper nanoclusters (black dots in Figure 3C and 3D) owing to the strong interactions of Cu NPs with the surface –COOH groups of the nanoporous polymer. TEM images of Cu-**A** are given in Figure 4A–4C, indicating that spherical Cu NPs with black color were uniformly distributed throughout the surface of the material and also incorporated inside the carbonaceous matrix. Its particle size range is from 4.7 nm to 5.3 nm. The morphology of Cu-**B** was further confirmed by FE-SEM images (Figure 4D–4F), showing uniform formation of sea-urchin-like copper-polymer nanostructures. It could be assumed that the physical phenomena involved for the development of this type of special morphology are (i) electrostatic interaction between carboxylic acid group and Cu^2+^ ions at the polymer surface to generate one-dimensional (1D) needles and (ii) diffusion of copper-polymer nanoclusters at the surface, which undergo the self-assembly to develop three-dimensional (3D) urchin-like spheres through oriented attachment. Magnified FE-SEM image (Figure 4E) exhibits that these nanostructures are composed of very thin needles. Thus, the formation mechanism can be explained on the basis of needle-to-urchin spheres, and polymer DVAC-1 has a definite role for the development of this special sea-urchin-like morphology^35^,^36^. EDX (energy dispersive X-ray) pattern and elemental mapping (Figure S1) of FE-SEM images prove the presence of C, O and Cu elements in the Cu-**B** nanocatalyst. EDX pattern and elemental mapping of each particular sea urchin nanostructures undoubtedly indicate that these nanostructures are composed of not only copper element but also C and O elements from the DVAC-1 polymer. It is expected that the unique nanostructures of Cu-**B** with high surface area could provide high catalytic performance for carrying out catalytic reactions.

### Catalytic Performance of Cu^0^ NP Based Nanocatalysts

**Figure 5:**
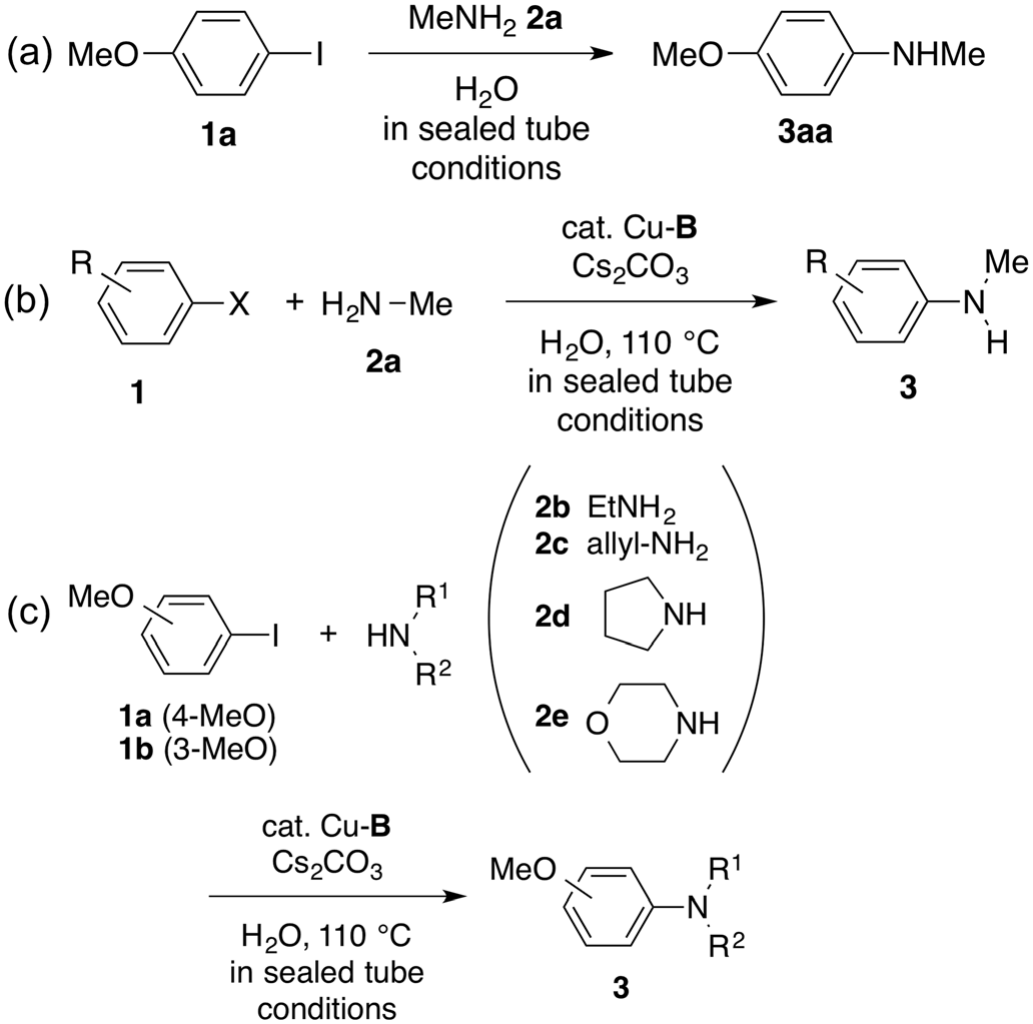
(a) Ullmann coupling of 4-iodoanisole (**1a**) with methylamine (**2a**). (b) Ullmann coupling of various aryl halides (**1**) with methylamine (**2a**). (c) Ullmann amination of iodoanisoles (**1a**) and (**1b**) with amines (**2**).

We then employed the newly prepared Cu^0^ NP based nanocatalysts Cu-**A** and Cu-**B** for the Ullmann coupling of aryl halides with amines. The reaction is shown in Figure 5. Our study began with the reaction of 4-iodoanisole (**1a**, 1.0 mmol) with methylamine (**2a**, 40% aqueous solution, 2.5 mL, 28.9 mmol) in the presence of Cs_2_CO_3_ (2 equiv) and the Cu-**A** nanocatalyst (20 mg, 1.38 mol% for Cu) in a sealed tube at 110°C (oil bath). The Cu content in the Cu-**A** catalyst is 0.690 mmol g^−1^, measured by inductive coupled plasma-mass spectroscopy (ICP-MS). The catalytic conversion could be observed and 4-methoxy-*N*-methylaniline (**3aa**) was isolated in 64% yield after 24 h (Table 1, entry 1). On the other hand, by employing the Cu-**B** catalyst (20 mg, 1.35 mol% for Cu) in the coupling reaction under otherwise identical reaction conditions, the yield of **3aa** could be improved to 76% and the reaction finished within only 3h (Table 1, entry 2). The Cu content in the Cu-**B** catalyst is 0.675 mmol g^−1^, determined by ICP-MS. The catalytic Ullmann coupling using Cu-**B** was also effective in large-scale reaction (10 mmol) with a consistent yield of 80% (Table 1, entry 3). The reaction with K_2_CO_3_ as the base instead of Cs_2_CO_3_ lowered the yield of **3aa** to 53% (Table 1, entry 4), while no reaction was observed when K_3_PO_4_ or Et_3_N was employed (Table 1, entries 5 and 6). In addition, the reaction did not take place in the presence of *N*,*N*-dimethylmethanamide or dimethyl sulfoxide as the solvent (Table 1, entries 7 and 8). We also checked the catalytic activity of Cu-**B** at room temperature (25°C) in the presence of Cs_2_CO_3_, and no conversion of the product was observed (Table 1, entry 9). We then conducted the Ullmann amination using bare Cu^0^ NPs as the catalyst (Table 1, entry 10)^37^. In this case, it took 24 h to complete the reaction and only 50% yield of **3aa** was obtained, suggesting that nanoporous polymer and unique sea-urchin nanostructure of Cu-**B** play a crucial role in the reaction efficiency. An additional control experiment for Ullmann coupling was carried out using Cu^0^ NP-loaded carbon black catalyst (Cu^0^-carbon black, synthesized by following a same procedure), showing the reaction yield of 50% after 24 h (Table 1, entry 11). The Cu loading in Cu^0^-carbon black catalyst is 0.695 mmol g^−1^, determined by ICP-MS. In order to examine main catalytically active site for this catalytic reaction, we performed the reaction using nanoporous polymer DVAC-1 without Cu. After 24 h of the reaction, no product conversion was observed (Table 1, entry 12), suggesting that the copper component is the major catalytically active site responsible to the catalytic reaction.

By using Cu-**B** as the catalyst, we next examined the substrate scope of this catalytic Ullmann coupling by employing various aryl halides **1** with methylamine **2a** (Table 2 and Figure 5). With *meta*- and *ortho*-iodoanisoles **1b** and **1c**, the corresponding anilines **3ba** and **3ca** could be prepared in 86% and 71% yields, respectively (Table 2, entries 1 and 2). Similarly, 4-iodotoluene (**1d**) and 1-chloro-4-iodobenzene (**1e**) could be transformed into the corresponding anilines **3da** and **3ea** in good yields (Table 2, entries 3 and 4). We tested several aryl bromides such as **1f** and **1g** as the coupling partners with methylamine (**2a**), delivering the corresponding anilines **3fa** ( = **3ba**) and **3ga** in moderate yields (Table 2, entries 5 and 6). The scope of amines **2** for the Ullmann amination with iodoanisoles **1a** and **1b** (Table 3 and Figure 5) was also examined. The reactions were smoothly performed with ethylamine (**2b**), allylamine (**2c**), and pyrrolidine (**2d**) in 12–18 h, affording the corresponding anilines in good yields (Table 3, entries 1–6), while the reaction with morpholine (**2e**) resulted in a moderate yield of the coupling product (Table 3, entry 7).

### Recyclability and Reusability of Cu-B Nanocatalyst

In order to investigate heterogeneous nature of the Cu-**B** nanocatalyst, hot filtration test and leaching test were carried out. These experimental results undoubtedly demonstrated that there was no leaching of copper from the nanocatalyst during the course of reaction and the nanocatalyst was indeed heterogeneous in nature. To evaluate the recyclability and reusability of the Cu-**B** nanocatalyst, we performed the Ullmann *N*-arylation of 4-iodoanisole (**1a**) with methylamine (**2a**) under the optimized reaction conditions. After the completion of the reaction, the reaction mixture was centrifuged and filtered. The recovered Cu-**B** nanocatalyst was washed by sufficient amount of water, ethyl acetate, and acetone. Finally, the nanocatalyst was dried in an oven at 80°C before the use for next catalytic cycle. The nanocatalyst could be reactivated in a very easy way. No addition of acid, base, and special reagent, and no special treatment (such as calcinations at high temperature) were needed during the reactivation. In every cycle, the nanocatalyst recovery was almost quantitative.

**Figure 6:**
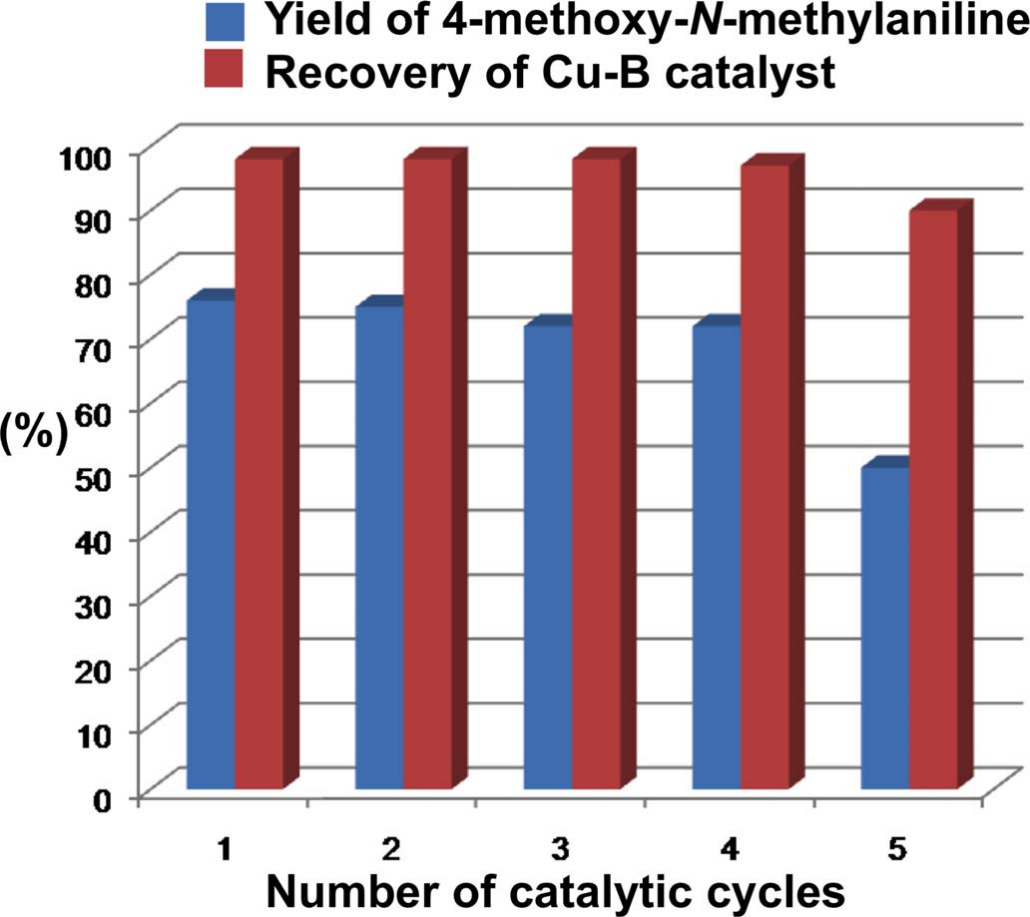
Recyclability and reusability of the Cu-B nanocatalyst for the Ullmann amination. Reaction conditions: 4-iodoanisole (1a, 1 mmol, 234 mg), methylamine (2a, 40 wt % aqueous solution, 2.5 mL, 28.9 mmol), Cs_2_CO_3_ (2 mmol, 651 mg), Cu-**B** (20 mg, 1.35 mol% for Cu), temperature 110°C, and time 3 h.

Figure 6 shows that the Cu-**B** nanocatalyst could be effectively recycled and reused up to five consecutive catalytic cycles, but an obvious decrease in the product yield was observed at the 5^th^ cycle. The drop of product yield at the 5^th^ catalytic cycle may be due to the clogging of nanopores by reagents. To identify the possible reagents responsible for the clogging of nanopores, EDX analysis and elemental mapping (Figure S2) of reused Cu-**B** nanocatalyst after the 5^th^ catalytic cycle followed by thorough washing with water, ethyl acetate and acetone were carried out. Surprisingly, The results reveal that a significant amount of Cs(I) ion from Cs_2_CO_3_ was adsorbed and trapped into the nanopores, limiting the diffusion of the organic substrates into the nanopores, and thus reducing the catalytic activity^38^. Unfortunately, thorough washing did not improve the catalytic yield at the 5^th^ catalytic cycle. Washing with organic solvents could remove unreacted organic substrates, but that with water cannot completely remove adsorbed Cs(I) ion from nanopores owing to hydrophobic nature of the nanoporous polymer. We also analyzed the filtrate after the recovery of the nanocatalyst by utilizing AAS (atomic absorption spectroscopy) technique in order to check if any Cu leaching took place from the nanocatalyst into the filtrate. The results indicate that the Cu content in the filtrate was below the detection limit of the instrument, and the filtrate was completely colorless.

### Characterization of Reused Cu-B Nanocatalyst

After the fifth catalytic cycle, we characterized the reused Cu-**B** nanocatalyst to confirm its mechanical stability. Figure S3 indicates that the nanocatalyst was stable during the course of catalytic reaction. Wide angle powder XRD pattern (Figure S3A) of the reused Cu-**B** nanocatalyst after the fifth catalytic cycle proves that the crystalline phase of the Cu^0^ NPs on the framework remains unchanged after the catalytic reaction. The XPS data (Figure S3B) of the reused Cu-**B** nanocatalyst suggest that the oxidation state of Cu remains unaltered after the Ullmann coupling. N_2_ adsorption/desorption isotherm analysis (Figure S3C) was also carried out for the reused Cu-**B** nanocatalyst after the 5th catalytic cycle. The BET surface area and corresponding pore volume of the reused Cu-**B** nanocatalyst are 180 m^2^g^−1^ and 0.432 ccg^−1^, respectively. Substantial decreases in the BET surface area and pore volume of the reused Cu-**B** nanocatalyst after the 5th catalytic cycle as compared with the fresh Cu-**B** nanocatalyst indicate that the clogging of nanopores took place, hindering the adsorption of nitrogen into the nanoporous channels.

In order to check mechanical stability of reused Cu-**B** nanocatalyst after five catalytic cycles, ^13^C solid state CP MAS (cross-polarization magic angle spinning) NMR analysis was carried out (Figure S3D), demonstrating that the structural integrity of organic functional groups in the nanoporous polymer backbone was retained. The TEM analysis (Figure S4) of the Cu-**B** nanocatalyst after each catalytic cycle shows no obvious aggregation of NPs, further indicating its high stability during the catalytic reactions. The Cu leaching test was also carried out. The Cu content of the nanocatalyst after the 5th catalytic cycle was found to be 0.669 mmolg^−1^, which is still comparable to that of the fresh catalyst (0.675 mmolg^−1^). This observation proves that no Cu leaching occurred during the course of reactions.

## Discussion

In this work, a novel nanoporous polymer DVAC-1 was designed and synthesized using acrylic acid through a facile one-pot hydrothermal technique, which was employed as a platform for the encapsulation and deposition of Cu^0^ NPs to achieve nanoporous polymer supported Cu^0^ heterogeneous nanocatalyst (Cu-**B**). Using the easy and efficient approach, the developed carboxylic acid functionalized polymer network could act as a strong anchoring agent for the coordination with metallic copper. The white color nanoporous polymer DVAC-1 contains 1.01 mmol/g carboxylic acid sites, determined by employing NH_3_-TPD profile (Figure 2F). Deposited Cu^0^ NPs with 1D needle-like structures self-assembled to deliver 3D sea-urchin-like morphology, which was confirmed by FE-SEM images. The deposition of Cu^0^ NPs on the external surface as well as in the nanoporous channel of the polymer was proven by the TEM images and N_2_ adsoprtion/desorption isotherms (Figures 2 and 3). Higher catalytic activity of the Cu-**B** nanocatalyst as compared with the Cu-**A** nanocatalyst is due to the synergetic effect of high surface area and unique hierarchical sea-urchin-like morphology. The most exposed crystallographic facets play a crucial role to tune catalytic property of the nanocatalyst. The adsorption followed by the activation of the reactant molecules as well as the desorption of the products are significantly assisted by the surface atomic arrangement of the most exposed crystalline planes. In other words, the most exposed crystalline planes of the nanocatalyst noticeably alter the catalytic property. From a catalysis point of view, high density of atomic steps, ledges, kinks and dangling bonds on high-index crystalline facets facilitates the improvement of catalytic activity. This phenomenon could be referred as morphology-dependent nanocatalysis. The special sea-urchin-like nanostructures of Cu-**B** could allow multiple reflections, so that catalytically active Cu sites interact preferably with the organic aryl halides by exposing their more reactive (111) crystalline facet. Thus, it is clear that the morphology and surface area play an important role in the enhancement of catalytic activity of the Cu-**B** nanocatalyst (Table 1, entry 2)^39^,^40^. In addition, higher catalytic activity of Cu-**B** nanocatalyst as compared with bare Cu^0^ NPs and Cu^0^-carbon black catalyst may be due to the synergetic effects of nano-confinement and electron donation of nanoporous polymer DVAC-1. The organic functional groups could donate π electrons to the surface of Cu^0^ NPs, making the surface more active for Ullmann amination reaction. After the reuse of the Cu-**B** nanocatalyst for five catalytic cycles, it was proven that the nanocatalyst was mechanically stable and no Cu leaching took place. The Cu-**B** nanocatalyst becomes more economic as compared with commercially available black copper powder, which requires 5 mol% Cu loading^41^.

In conclusion, we have successfully developed the Cu^0^ NP embedded nanoporous polymer (Cu-**B**) as a heterogeneous catalyst, which exhibits excellent catalytic activity for the Ullmann coupling of aryl halides with primary and secondary amines to produce *N*-aryl amines in aqueous solution. All the catalytic reactions have been conducted in a low loading of Cu (1.35 mol%). The high BET surface area and unique sea-urchin-like nanostructure of the Cu-**B** nanocatalyst make it more catalytically efficient as compared with its analogue Cu-**A** nanocatalyst. Cu-**B** nanocatalyst also exhibits better catalytic performance as compared with Cu^0^-carbon black catalyst and bare Cu^0^ NPs. The Cu-**B** nanocatalyst could be recycled and reused for five consecutive reaction cycles without obvious Cu leaching during the reactions. Thus, the developed heterogeneous Cu^0^ catalyst may find its way towards more practical catalytic applications.

## Methods

### Characterization techniques

Powder X-ray diffraction (XRD) patterns were recorded on a Bruker D-8 Advance SWAX diffractometer operated at 40 kV voltage and 40 mA current. The instrument was calibrated with a standard silicon sample using Ni-filtered Cu K_α_ (α = 0.15406 nm) radiation. Nitrogen adsorption/desorption isotherms were obtained using a Quantachrome Autosorb 1C at 77 K. Prior to gas adsorption, the sample was degassed at 373 K for 4 h. A JEOL JEM 6700F field emission scanning electron microscope (FE-SEM) was used for determining the morphology of the samples. Transmittance electron microscopic (TEM) images were recorded on a JEOL JEM 2010 transmission electron microscope. Cu loading in the sample was estimated by using a Perkin-Elmer Optima 2100 DV Inductive Coupled Plasma Mass Spectroscopy (ICP-MS). Raman spectra on cleaned silicon substrate were measured using a Raman microscope (LabRAM HR, Horiba Yvon). The excitation wavelength of the irradiating light was 632.8 nm (He-Ne Laser, Melles Griot, laser excitation 0.1 mW) and signals were collected by using ×50 objective lens. X-ray photoelectron spectroscopy (XPS) analysis was carried out by a SPECS I3500 plus spectrometer using Mg X-ray source.^1^H NMR (400 MHz) spectra were recorded on a Bruker Avance 400 spectrometers in CDCl_3_ by using CDCl_3_ (for 1H, δ = 7.26) as the internal standard unless otherwise stated. ^13^C NMR spectra were performed on a Bruker Avance 400 spectrometer in CDCl_3_ using CDCl_3_ (for ^13^C, δ = 77.00) as the internal standard. The following abbreviations were used to explain the multiplicities: s = singlet, d = doublet, t = triplet, q = quartet, m = multiplet, and bs = broad singlet. Temperature programmed desorption (TPD) of ammonia from the sample was carried out using a thermal conductivity detector (TCD) in a Micromeritics Chemisorb 2720 instrument. For the experiment, the sample was first degassed in flow of He at a flow rate of 30 CC at 100°C for 2 h. Then, the sample was saturated with 10% NH_3_ in He at room temperature for 30 min. The excess NH_3_ was removed by flow of He (flow rate of 30 CC) for 45 min. TPD of ammonia was studied by heating from room temperature to 700°C at a temperature ramp of 10°C/min.

### Synthesis of Cu-A nanocatalyst

The Cu-A catalyst was synthesized by chelate-assisted co-assembly strategy using Cu(acac)_2_ as a source of copper. Soluble phenol formaldehyde resin precursor was developed according to previously reported procedure^42^. Poly(ethylene oxide)-*block*-poly(propylene oxide)-*block*-poly-(ethylene oxide) triblock copolymer Pluronic F127 (M_w_ = 12600, PEO_106_PPO_70_PEO_106_, 500 mg) was dissolved in ethanol (15 mL) with the addition of aqueous HCl (12 M, 0.825 g). After getting a clear solution at room temperature, phenolic resin (0.645 g) in ethanol (5 mL) was added dropwise^43^, which was stirred at room temperature for 15 min. Then, Cu(acac)_2_ (108 mg, 0.412 mmol) in ethanol (10 mL) was added dropwise into the above solution and resulted reaction mixture was stirred at room temperature for another 6 h. The mixture was poured into a petridish and evaporated by keeping in a fume hood for 24 h. The obtained sticky film was subjected to thermocuring at 100°C for 24 h. The deep brown color solid polymer was scratched off and pyrolyzed in a tube furnace under N_2_ atmosphere at 600°C for 6 h. Finally, a black color material was prepared, which was designated as the Cu-**A** nanocatalyst.

### Synthesis of nanoporous polymer DVAC-1

DVAC-1 nanoporous polymer was prepared *via* non-aqueous polymerization of a mixture of divinylbenzene and acrylic acid under hydrothermal condition using azobisisobutyronitrile (AIBN) as a radical initiator. In a typical synthesis procedure, divinylbenzene (5.99 mmol, 781 mg) and acrylic acid (1.49 mmol, 108 mg) were mixed together in a round-bottomed flask containing acetone (15 mL). To the mixture, AIBN (0.152 mmol, 25 mg) was added and the resulted mixture was allowed to stir at room temperature under nitrogen atmosphere for 10 h. Then, the resultant mixture was hydrothermally treated in an autoclave at 120°C under static condition for 24 h. The final white color solid material DVAC-1 was isolated and dried in air.

### Synthesis of Cu-B nanocatalyst

The Cu-B nanocatalyst was developed from nanoporous polymer DVAC-1. DVAC-1 polymer (300 mg) was dispersed in NaOH solution (40 mL, 2 M) by stirring at room temperature followed by sonication at room temperature for about 2 h. Light yellowish color dispersion was obtained. Then, CuCl_2_•2H_2_O (98 mg, 0.574 mmol) was dissolved in water (5 mL), which was added dropwise into the above dispersion. The resulted mixture was allowed to stir at room temperature for about 10 h, leading to a color change from light blue to light grey. In this step, Cu^2+^ ions were attached to the carboxylate ions on the surface of nanoporous polymer *via* electrostatic interactions. Next, ethylene diamine (EDA, 2 mL) was added to this mixture, which was stirred at room temperature for 30 min. The Cu(EDA)_2_^2+^ complex was formed, indicated by the color change of the mixture from light grey to deep violet. After 1 h, aqueous solution of hydrazine hydrate (2 mL, 35 wt%) was added dropwise into this solution, which was heated to 80°C under nitrogen atmosphere for about 6 h. During this process, the color of the resulted mixture was changed into deep brown, indicating that Cu^0^ NPs were generated in one-step *in situ* reduction of Cu^2+^ ions. Then, the reaction mixture was cooled down to room temperature and centrifuged to afford the brownish material. The solid material was washed with ethanol three times followed by washing with acetone, and dried in a fridge drier to give brownish color solid Cu-**B**.

### Synthesis of bare Cu^0^ NPs

Bare Cu^0^ NPs were prepared using CuCl_2_•2H_2_O as copper source in the absence of DVAC-1 nanoporous polymer. CuCl_2_•2H_2_O (107 mg, 0.627 mmol) was dissolved in water (5 mL), affording a transparent green solution. NaOH solution (2 M, 5 mL) was added dropwise into the above solution to make the solution basic. EDA (2 mL) was added, which was allowed to stir at room temperature for 30 min. Then, aqueous solution of hydrazine hydrate (2 mL, 35 wt%) was added dropwise into the resulted mixture, which was heated to 80°C under nitrogen atmosphere for about 6 h. During this process, the color of the resulted mixture was changed into deep brown, indicating that Cu^0^ NPs were generated. Finally, the reaction mixture was cooled down to room temperature and centrifuged to afford the brownish Cu^0^ NPs^37^.

### Synthesis of Cu^0^-carbon black catalyst

Activated carbon black (300 mg) was dispersed in water (20 mL) by stirring at room temperature followed by sonication in an ultrasonication bath for about 6 h. Then, CuCl_2_•2H_2_O (103 mg, 0.6041 mmol) was dissolved in water (5 mL), which was added dropwise into the above dispersion. The resulted mixture was allowed to stir at room temperature for about 12 h, leading to a black dispersion. After that, aqueous solution of hydrazine hydrate (2 mL, 35 wt%) was added dropwise into this dispersion, which was heated to 80°C under nitrogen atmosphere for about 6 h. The resulted mixture was cooled down to room temperature, and the obtained black solid material was isolated by normal filtration followed by washing with ethanol. This black material was designated as Cu^0^-carbon black.

### General procedure for Ullmann N-arylation of aryl iodides 1 with methyl amine (2a) in water catalyzed by the Cu-B nanocatalyst

Aryl iodide **1** (1.0 mmol), Cu-**B** (20 mg, 1.35 mol% for Cu) and Cs_2_CO_3_ (2.0 mmol, 650 mg) dissolved in aqueous solution of MeNH_2_ **2a** (28.9 mmol, 2.5 mL, 40 wt%) were placed in a 25 mL sealed tube. The tube was capped tightly and subjected to heat at 110°C in an oil bath for the time referred. When the reaction was completed (monitored by TLC), the sealed tube was taken out from the oil bath and cooled down to room temperature. The reaction mixture was diluted with EtOAc (5 mL) and then centrifuged. The nanocatalyst was settled down and the biphasic mixture was taken into a separating funnel. The organic layer was collected in a conical flask and the aqueous layer was washed twice with EtOAc (2 × 30 mL). Combined organic layer was dried over anhydrous MgSO_4_ and then evaporated to give the crude mixture, which was purified by column chromatography using a mixture of hexane and EtOAc.

### Hot filtration test

Hot filtration test was conducted to confirm that the nanocatalysis was indeed heterogeneous. In this test, a mixture of Cu-**B** (20 mg), 4-iodoanisole (1 mmol) and aqueous methylamine (2.5 mL) was subjected to heat at 110°C for 1 h. The Cu-**B** nanocatalyst was then filtered off from the hot reaction mixture, and the *N*-arylation reaction in the filtrate was still monitored. No increase in conversion was observed in the filtrate.

### Leaching test

ICP-MS technique was applied in order to determine the content of Cu in fresh nanocatalyst before and after the reaction. AAS analysis was employed to make sure that no leaching of Cu occurs in the reaction mixture during the course of Ullmann amination reaction. After the catalytic reaction was over, the Cu-**B** nanocatalyst was separated from the reaction mixture with centrifugation technique and washed with acetone, and then the filtrate was subjected to evaporate to dryness. 30 wt % nitric acid was added to the filtrate, and the resulting mixture was adjusted to 5 wt%. AAS analysis of the filtrate indicates that no detectable amount of Cu was present in the filtrate. It should be noticed that the filtrate remains complete colorless. ICP-MS analysis of the reused nanocatalyst indicates that the Cu content decreased a little bit to 0.669 mmolg^−1^ after five catalytic cycles. This decrease is within the experimental error of chemical analysis. The experimental data clearly demonstrate that the Cu-**B** nanocatalyst is truly heterogeneous in nature, and there was no leaching of Cu from the Cu-**B** nanocatalyst during the course of reactions.

## Author Contributions

Y.L.Z. and J.M. have designed the research. A.B. and S.C. have designed methodology the Ullmann coupling catalytic reaction. J.M. has carried out the synthesis and characterization of Cu-nanocatalysts. A.B. and J.M. have carried out all the catalytic reactions, isolated the products and characterized the all products. All the authors discussed the results and commented on the manuscript.

## Supplementary Material

Supplementary Information Supplementary Information

## Figures and Tables

**Table 1:** Optimization of the reaction conditions for the Ullmann coupling of 4-iodoanisole (**1a**) and methylamine (**2a**)a)

Entry	Catalyst	Base	Temperature (°C)	Time (h)	Yield (%)b)	TON
1	Cu-A	Cs_2_CO_3_	110	24	64	46.3
2	Cu-B	Cs_2_CO_3_	110	3	76	56.2
3c)	Cu-B	Cs_2_CO_3_	110	3	80	59.2
4	Cu-B	K_2_CO_3_	110	24	53	39.2
5	Cu-B	K_3_PO_4_	110	24	NR	0
6	Cu-B	NEt_3_	110	24	NR	0
7d)	Cu-B	Cs_2_CO_3_	110	24	NR	0
8e)	Cu-B	Cs_2_CO_3_	110	24	NR	0
9f)	Cu-B	Cs_2_CO_3_	25	24	NR	0
10	Cu^0^ NPs	Cs_2_CO_3_	110	24	50	36.2
11	Cu^0^-Carbon Black	Cs_2_CO_3_	110	24	50g)	35.9
12	DVAC-1	Cs_2_CO_3_	110	24	NR	0

^a)^Reaction conditions: 4-iodoanisole (**1a**, 1 mmol, 234 mg), methylamine (**2a**, 40 wt % aqueous solution, 2.5 mL, 28.9 mmol) and inorganic base (2 mmol);

^b)^Isolated yields;

^c)^The reaction was conducted using **1a** (10 mmol), **2a** (40 wt % aqueous solution, 25 mL), and Cs_2_CO_3_ (20 mmol);

^d)^Methyl amine (40 wt% aqueous solution, 1 mL) and *N*,*N*-dimethylformamide (2 mL);

^e)^Methyl amine (40 wt% aqueous solution, 1 mL) and dimethyl sulfoxide (2 mL);

^f)^The reaction was performed at room temperature;

^g)^Determined by^1^H NMR spectra. NR = no reaction; TON = turn over number (mole of substrate converted per mole of active site).

**Table 2:** Scope on the Ullmann coupling with methylamine (**2a**)a)

Entry	1 (R, X)	Yield (%)b)	TON
1	**1b** (R = 3-MeO; X = I)	**3ba** 86% (16 h)	63.7
2	**1c** (R = 2-MeO; X = I)	**3ca** 71% (4 h)	52.6
3	**1d** (R = 4-Me; X = I)	**3da** 70% (3 h)	51.8
4	**1e** (R = 4-Cl; X = I)	**3ea** 73% (4 h)	54.0
5	**1f** (R = 3-MeO; X = Br)	**3fa** 51% (67%)c) (12 h)	49.6
6	**1g** (R = 3-Cl; X = Br)	**3ga** 51% (64%)c) (16 h)	49.4

^a)^Reaction conditions: aryl iodides **1** (1 mmol), methylamine (**2a**, 40 wt % aqueous solution, 2.5 mL, 28.9 mmol), Cs_2_CO_3_ (2 mmol, 651 mg), Cu-**B** (20 mg, 1.35 mol% for Cu), and temperature 110°C;

^b)^Isolated yields;

^c)^Yields based on the recovery of starting materials.

**Table 3:** Scope on the Ullmann amination with aryl iodides **1a** and **1b**a)

Entry	Aryl Iodides	Amines	Yield (%)b)	TON
1	**1a**	**2b**	**3ab** 63% (12 h)	46.6
2	**1b**	**2b**	**3bb** 68% (12 h)	50.3
3	**1a**	**2c**	**3ac** 64% (18 h)	47.4
4	**1b**	**2c**	**3bc** 64% (12 h)	47.4
5	**1a**	**2d**	**3ad** 74% (16 h)	54.8
6	**1b**	**2d**	**3bd** 68% (12 h)	50.3
7	**1a**	**2e**	**3ae** 41%, 60%c) (24 h)	30.7

^a)^Reaction conditions: 3- or 4-iodoanisole (1 mmol, 234 mg), amine (1 mL, 12.0–15.2 mmol), Cs_2_CO_3_ (2 mmol, 651 mg), H_2_O (2 mL), Cu-**B** (20 mg, 1.35 mol% for Cu), and temperature 110°C;

^b)^Isolated yields;

^c)^Conversion yield.
