# Supplementary material for: Cu0 Nanoparticles Deposited on Nanoporous Polymers: A Recyclable Heterogeneous Nanocatalyst for Ullmann Coupling of Aryl Halides with Amines in Water
Source: Sci Rep. 2015 Feb 6;5:8294. doi: 10.1038/srep08294 (PMC4319161; doi:10.1038/srep08294)
Supplement: Supplementary Information [file srep08294-s1.doc]

**Cu^0^ Nanoparticles Deposited on Nanoporous Polymers: A Recyclable Heterogeneous Nanocatalyst for Ullmann Coupling of Aryl Halides with Amines in Water**

John Mondal,^1^ Anup Biswas,^1^ Shunsuke Chiba^1^ & Yanli Zhao^1,2^

^1^ Division of Chemistry and Biological Chemistry, School of Physical and Mathematical Sciences, Nanyang Technological University, 21 Nanyang Link, Singapore 637371

^2^ School of Materials Science and Engineering, Nanyang Technological University, Singapore 639798


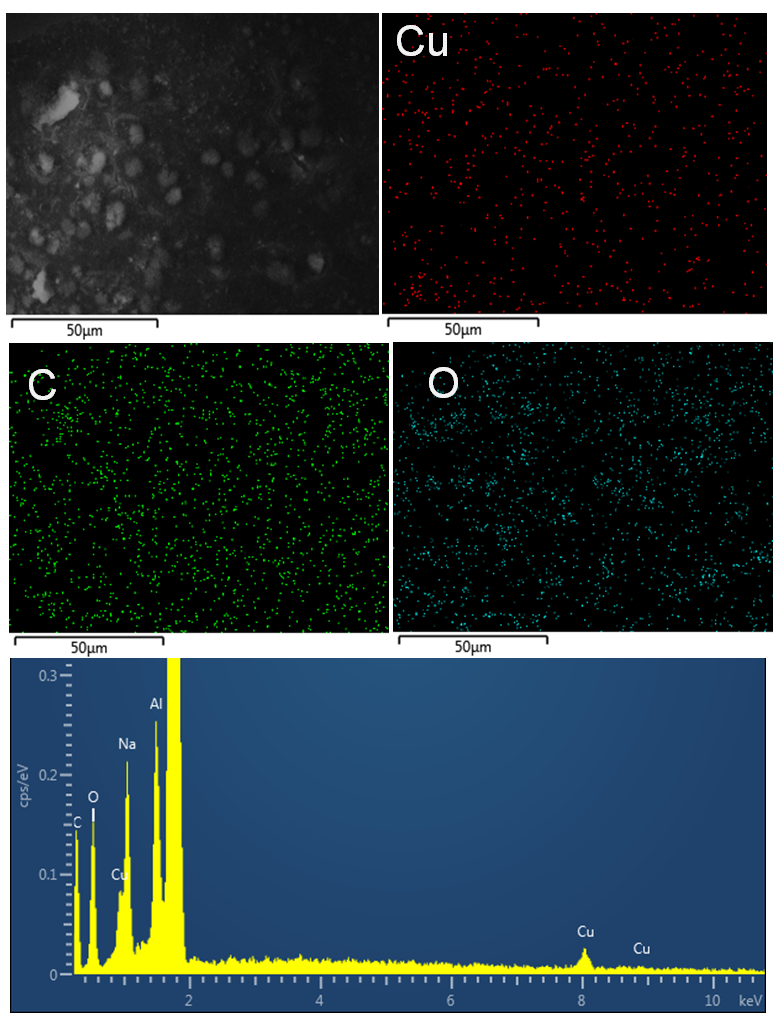


Figure S1: FE-SEM image of Cu-**B** catalyst as well as C, O and Cu elemental mapping and corresponding EDX spectral analysis.


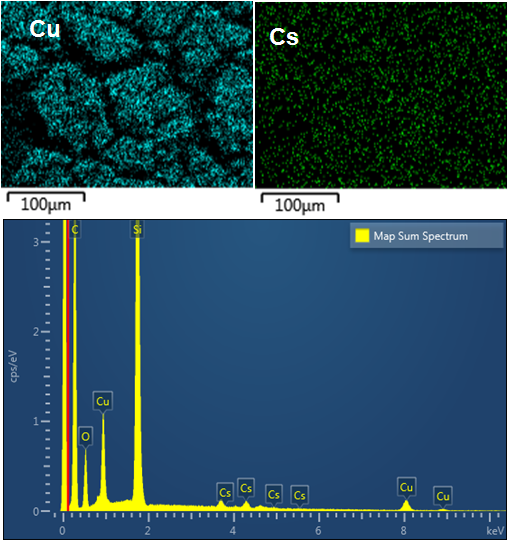


Figure S2: Elemental mapping of Cs and Cu and corresponding EDX spectral analysis for reused Cu-**B** catalyst after the 5^th^ catalytic cycle.


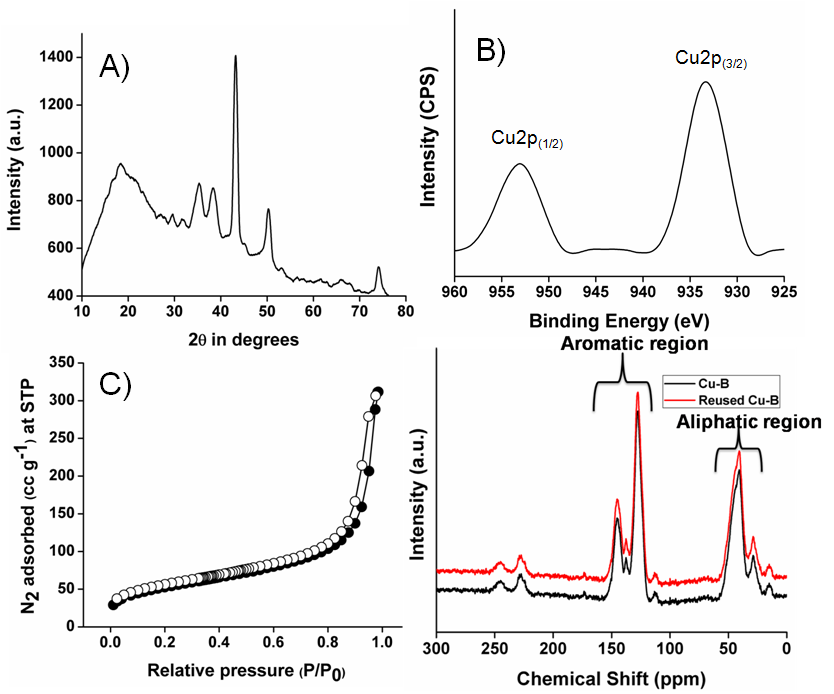


Figure S3: A) Wide angle powder XRD pattern, B) High resolution XPS spectrum, C) N_2_ adsorption/desorption isotherms, and D) ^13^C CP MAS NMR spectra of the reused Cu-**B** catalyst after the 5^th^ catalytic cycle.


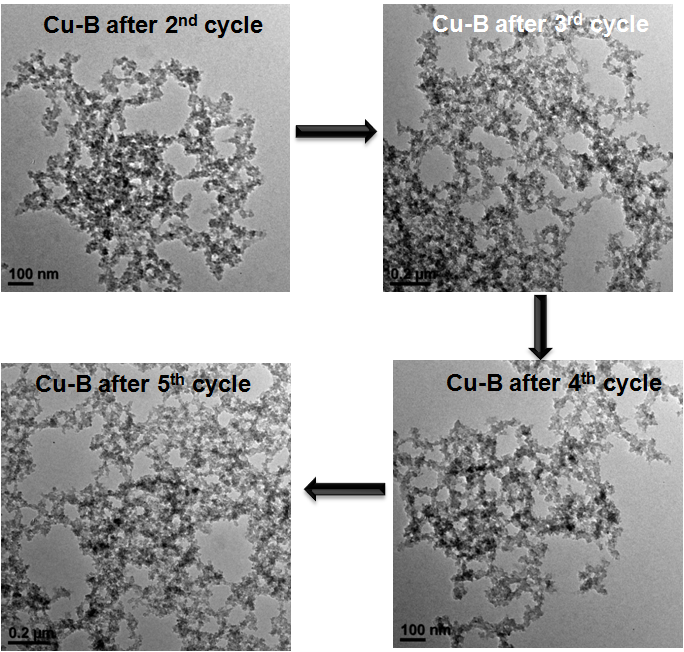


Figure S4: TEM images of the reused Cu-**B** catalyst for the Ullmann amination after each catalytic cycle.

**^1^H and ^13^C NMR data of all products referred in Tables 1, 2 & 3**

**4-Methoxy-*N*-methylaniline (3aa):**^1^ ^1^H NMR (CDCl_3_, 400 MHz) δ 2.82 (3H, s), 3.45 (NH, bs), 3.78 (3H, s), 6.61 (d, *J* = 8.8 Hz, 2H), 6.84 (d, *J*= 8.8 Hz, 2H)**;** ^13^C NMR (CDCl_3_, 100 MHz) δ 31.4, 55.7, 113.5, 114.8, 143.6, 151.9.

**3-Methoxy-N-methylaniline (3ba):**^2^ ^1^H NMR (CDCl_3_, 300 MHz) δ2.84 (s, 3H), 3.74 (bs, 1H), 3.80 (s, 3H), 6.18-6.19 (m, 1H), 6.24-6.33 (m, 2H), 7.12 (t, *J* = 8.1 Hz, 1H)**;** ^13^C NMR (CDCl_3_,75 MHz) δ 30.6, 55,0, 98.2, 102.2, 105.6, 129.8, 150.7, 160.8.

**2-Methoxy-*N*-methylaniline (3ca):**^3^ ^1^H NMR (CDCl_3_, 400 MHz) δ 2.92 (s, 3H), 3.89 (s, 3H), 4.03 (bs, NH), 6.67-6.69 (m, 1H), 6.73-6.77 (m, 1H), 6.82-6.84 (m, 1H), 6.97-7.00 (m, 1H)**;** ^13^C NMR (CDCl_3_,100 MHz) δ 30.4, 55.4, 109.3, 109.4, 116.3, 121.3, 121.4, 139.5, 147.0.

***N*,4-Dimethylaniline (3da):**^3^ ^1^H NMR (CDCl_3_, 300 MHz) δ 2.32 (s, 3H), 2.87 (s, 3H), 3.49 (bs, NH), 6.61 (d, *J* = 8.2 Hz, 2H), 7.08 (d, *J* = 8.2 Hz, 2H)**;** ^13^C NMR (CDCl_3_,75 MHz) δ 20.3, 31.0, 112.5, 126.3, 129.6, 147.1.

**4-Chloro-*N*-methylaniline (3ea):**^4^ ^1^H NMR (CDCl_3_, 300 MHz) δ 2.81 (s, 3H), 3.69 (bs, NH), 6.54-6.58 (m, 2H), 6.89-6.96 (m, 2H)**;** ^13^C NMR (CDCl_3_,75 MHz) δ 31.2, 113.0, 113.1, 115.4, 115.6.

**3-Chloro-*N*-methylaniline (3ga):**^4^ ^1^H NMR (CDCl_3_, 400 MHz) δ 2.82 (s, 3H), 6.45-6.48 (m, 1H), 6.56-6.57 (m, 1H), 7.07 (t, *J* = 8.0 Hz, 1H)**;** ^13^C NMR (CDCl_3_,100 MHz) δ 30.5, 110.8, 111.9, 117.0, 130.1.

***N*-Ethyl-4-methoxyaniline (3ab):**^5^ ^1^H NMR (CDCl_3_, 300 MHz) δ 1.27 (t, *J* = 7.2 Hz, 3H), 3.15 (q, *J* = 7.2 Hz, 2H), 3.55 (bs, NH), 3.79 (s, 3H), 6.69 (d, *J* = 9.2 Hz, 2H), 6.83 (d, *J* = 9.2 Hz, 2H)**;** ^13^C NMR (CDCl_3_, 75 Hz) δ 15.0, 39.6, 55.8, 114.3, 114.9, 142.7, 152.2.

***N*-Ethyl-3-methoxyaniline (3bb):**^6^ ^1^H NMR (CDCl_3_, 300 MHz) δ 1.26 (t, *J* = 7.2 Hz, 3H), 3.16 (q, *J* = 6.9 Hz, 2H), 3.59 (s, 3H), 3.79 (s, 3H), 6.18-6.19 (m, 1H), 6.26-6.31 (m, 2H), 7.10 (t, *J* = 7.8 Hz, 1H)**;** ^13^C NMR (CDCl_3_¸75 MHz) δ 14.8, 38.4, 55.0, 98.6, 102.2, 105.9, 129.9, 149.8, 160.8.

***N*-Allyl-4-methoxyaniline (3ac):**^7^ ^1^H NMR (CDCl_3_, 300 MHz) δ 3.45 (bs, NH), 3.73-3.75 (m, 2H), 3.76 (s, 3H), 5.18 (d, *J* = 10.2 Hz, 1H), 5.29 (d, *J* = 17.1 Hz, 1H), 5.92-6.05 (m, 1H), 6.62 (d, *J* = 9.0 Hz, 2H), 6.81 (d, *J* = 9.0 Hz, 2H)**;** ^13^C NMR (CDCl_3_,75 MHz) δ 47.6, 55.8, 114.3, 114.9, 116.1, 135.9, 142.3, 152.2.

***N*-Allyl-3-methoxyaniline (3bc):**^8^ ^1^H NMR (CDCl_3_, 300 MHz) δ 3.80-3.75 (m, 5H), 3.86 (bs, 1H), 5.21 (d, 1H, *J* = 12.0 Hz), 5.32 (d, *J* = 18.0 Hz, 1H), 5.92-6.04 (m, 1H), 6.22-6.34 (m, 3H),7.12 (t, *J* = 7.8 Hz, 1H)**;** ^13^C NMR (CDCl_3_,75 MHz) δ 160.7, 149.4, 135.5, 116.1, 106.0, 102.5, 98.9, 54.9, 46.4.

**1-(4-Methoxyphenyl)pyrrolidine (3ad):**^9^ ^1^H NMR (CDCl_3_, 300 MHz) δ 1.97-2.02 (m, 4H), 3.22-3.26 (m, 4H), 3.76 (s, 3H), 6.55 (d, *J* = 9.0 Hz, 2H), 6.85 (d, *J* = 9.0 Hz, 2H)**;** ^13^C NMR (CDCl_3_,75 Hz) δ 25.4, 48.2, 56.0, 112.6, 115.0, 143.2, 150.8.

**1-(3-Methoxyphenyl)pyrrolidine (3bd):**^10^ ^1^H NMR (CDCl_3_, 300 MHz) δ 2.00-2.06 (m, 4H), 3.30-3.35 (m, 4H), 3.85 (s, 3H), 6.17-6.18 (m, 1H), 6.24-6.32 (m, 2H), 7.19 (d,*J* = 8.2 Hz, 2H)**;** ^13^C NMR (CDCl_3_, 75 MHz) δ 25.5, 47.7, 55.1, 98.0, 100.5, 105.0, 129.8, 149.3, 160.8.

**1-(4-Methoxyphenyl)morpholine (3ae):**^11^ ^1^H NMR (CDCl_3_, 300 MHz) δ 3.05-3.07 (m, 4H), 3.77 (s, 3H), 3.85-3.87 (m, 4H), 6.84-6.91 (m, 4H); ^13^C NMR (CDCl_3_, 75 MHz) δ 50.8, 55.5, 67.0, 114.5, 117.8, 145.6, 153.9.

References:

| 1 | Jiao, J. *et al*. A facile and practical copper powder-catalyzed, organic solvent- and ligand-free Ullmann amination of aryl halides. *J. Org. Chem*. **76**, 1180-1183 (2011). |
| --- | --- |
| 2 | Hirayama, T. *et al*. Synthesis of a new bifunctionalised fluorescent label and physical properties of the bound form on model peptide of troponin C. *Org. Biomol. Chem.* **5**, 2040-2045 (2007). |
| 3 | González, I. *et al*. Selective monomethylation of anilines by Cu(OAc)_2_-promoted cross-coupling with MeB(OH)_2_**.** *Org. Lett.* **11**, 1677-1680 (2009). |
| 4 | Rani, A. U. *et al*. FT-IR, FT-Raman, NMR spectra and DFT calculations on 4-chloro-N-methylaniline. *Spectrochim. Acta.* A **75**, 1523-1529 (2010). |
| 5 | Lyle, R. E. & [Troscianiec](http://pubs.acs.org/action/doSearch?action=search&author=TROSCIANIEC%2C+HENRY+J.&qsSearchArea=author), H. J. Molecular rearrangements. Vi. the rearrangement of oximes on reduction with lithium aluminum hydride. *J. Org. Chem.* **20** 1757-1760 (1955). |
| 6 | Sydnes, M. O. & Isobe, M. One-pot reductive monoalkylation of nitro aryls with hydrogen over Pd/C. *Tetrahedron Lett*. **49**, 1199-1202 (2008). |
| 7 | Brucelle, F. & Renaud, P. Synthesis of indolines, indoles, and benzopyrrolizidinones from simple aryl azides. *Org. Lett*. **14**, 3048-3051 (2012). |
| 8 | Anderson, W. K. & Lai, G. Boron trifluoride-diethyl ether complex catalyzed aromatic amino-claisen rearrangements. *Synthesis* 1287-1290 (1995). |
| 9 | He, H. *et al*. N-Heterocyclization of primary amines with dihalides using microreactors. *Synth. Commun*. **42**, 2512-2525 (2012). |
| 10 | Liu, Z. & Larock, R. C. Facile *N*-arylation of amines and sulfonamides and *O*-arylation of phenols and arenecarboxylic acids. *J. Org. Chem.* **71**, 3198-3209 (2006). |
| 11 | Nathel, N. F. F. *et al*. Nickel-catalyzed amination of aryl chlorides and sulfamates in 2-methyl-THF. *ACS Catal*. **4**, 3289−3293 (2014). |
